# Liver Growth Factor Induces Glia-Associated Neuroprotection in an In Vitro Model of Parkinson´s Disease

**DOI:** 10.3390/brainsci10050315

**Published:** 2020-05-22

**Authors:** Rafael Gonzalo-Gobernado, Diana Reimers, María José Casarejos, Lucía Calatrava Ferreras, Manuela Vallejo-Muñoz, Adriano Jiménez-Escrig, Juan José Diaz-Gil, Gonzalo M. Ulzurrun de Asanza, Eulalia Bazán

**Affiliations:** 1 Servicio de Neurobiología, Instituto Ramón y Cajal de Investigación Sanitaria (IRYCIS), 28034 Madrid, Spain; diana.reimers@hrc.es (D.R.); m.jose.casarejos@hrc.es (M.J.C.); luciacalatrava@gmail.com (L.C.F.); manuela.vmqm@gmail.com (M.V.-M.); jjdiazgil@yahoo.es (J.J.D.-G.); gonzalo.munoz@hrc.es (G.M.U.d.A.); 2 National Centre for Biotechnology (CNB), CSIC, 28049 Madrid, Spain; 3 Servicio de Neurología, Hospital Ramón y Cajal, 28034 Madrid, Spain; adriano.jimenez@hrc.es

**Keywords:** liver growth factor, LGF, Parkinson´s disease, glial cultures, neuroprotection, 6-hydroxy-dopamine, microglia, TNF-alpha, mesencephalic neurons

## Abstract

Parkinson’s disease is a neurodegenerative disorder characterized by the progressive death of dopaminergic (DA) neurons in the substantia nigra (SN), which leads to a loss of the neurotransmitter dopamine in the basal ganglia. Current treatments relieve the symptoms of the disease, but none stop or delay neuronal degeneration. Liver growth factor (LGF) is an albumin–bilirubin complex that stimulates axonal growth in the striatum and protects DA neurons in the SN of 6-hydroxydopamine-lesioned rats. Our previous results suggested that these effects observed in vivo are mediated by microglia and/or astrocytes. To determine if these cells are LGF targets, E14 (embryos from Sprague Dawley rats of 14 days) rat mesencephalic glial cultures were used. Treatment with 100 pg/mL of LGF up-regulated the mitogen-activated protein kinases (MAPKs) extracellular signal-regulated kinases 1/2 (ERK1/2) and the cyclic AMP response element binding protein (CREB) phosphorylation in glial cultures, and it increased the microglia marker Iba1 and tumor necrosis factor alpha (TNF-alpha) protein levels. The treatment of E14 midbrain neurons with a glial-conditioned medium from LGF-treated glial cultures (GCM-LGF) prevented the loss of DA neurons caused by 6-hydroxy-dopamine. This neuroprotective effect was not observed when GCM-LGF was applied in the presence of a blocking antibody of TNF-alpha activity. Altogether, our findings strongly suggest the involvement of microglia and TNF-alpha in the neuroprotective action of LGF on DA neurons observed in vitro.

## 1. Introduction

Parkinson disease (PD) is a neurodegenerative disorder characterized by a series of motor symptoms like tremors, slowness of movement, rigidity, and bradykinesia [1]. The neuropathological feature of PD is the progressive death of dopaminergic neurons in the substantia nigra (SN), which leads to a loss of the neurotransmitter dopamine in the striatum, the nucleus that receives axonal projections from the SN [2]. Though the cause of the disease remains difficult to elucidate, different mechanisms such as dopamine-dependent oxidative stress, mitochondrial dysfunction, excitotoxicity, and proteasomal dysfunction have been associated with the loss of these neurons. In addition, several studies have demonstrated the involvement of neuroinflammatory processes in the degeneration of neurons in the SN [3,4,5].

Current treatments relieve the symptoms of the disease, but none stop or delay neuronal degeneration [1]. Levodopa (L-DOPA) is the standard treatment due to its effectiveness in controlling the symptoms of PD, but it is associated with long-term motor complications, such as motor fluctuations and dyskinesia [6]. Neurotrophic factors are molecules that promote the survival, differentiation, and maintenance of neurons during development and in the adult nervous system [7]. These factors are potential tools for the treatment of neurodegenerative diseases, including PD. Thus, in experimental models of PD glial-derived neurotrophic factor (GDNF) or brain-derived neurotrophic factor (BDNF) were able to protect damaged neurons in the SN [8]. However, the fact that, to be effective, these factors must be directly applied into the brain, and in many cases the use of modified cells is necessary [9], makes their application to the clinic quite difficult.

Liver growth factor (LGF) is a hepatic mitogen purified in 1986 by Dr. Diaz-Gil group. This 64 kDa factor is able to stimulate DNA synthesis and increase the proliferation and activity of the amino acids transport system of hepatocytes [10]. Diaz-Gil and colleagues demonstrated that LGF is an albumin–bilirubin complex whose concentration is virtually undetectable in rat and human serum from healthy individuals, but it increases significantly in the process of liver damage [11,12]. Several studies have shown that LGF increases the proliferation of different cell types and promotes regeneration of damaged tissues, including the brain [13,14,15]. Thus, the intracerebral administration of LGF to hemiparkinsonian rats was found to be able to stimulate axonal growth in the striatum, as well as to improve rotational behavior stimulated by apomorphine [16]. Moreover, LGF was found to promote the proliferation and migration of neural progenitors from the forebrain subventricular zone [17], as well as to improve the viability, differentiation, and integration of neural stem cell grafts into the host tissue [18]. More recently, we demonstrated that intraperitoneally applied LGF was also able to stimulate axonal growth in the striatum in order to increase the number of dopaminergic neurons in the damaged SN and to improve rotational behavior stimulated by apomorphine in an experimental model of Parkinson’s disease [19]. 

An important question to be determined is the type of target cell and molecular effectors that mediate the actions of the LGF in PD. Glial cells synthesize and release a number of factors that play important roles in the survival of neurons, remyelination, and the formation of new synapses [20,21,22]. In hemiparkinsonian rats, LGF treatment has been found to stimulate glial cells, specifically promoting the activation of microglia in the striatum [16,17,19]. Though activated microglia has been associated with the pathogenesis of several neurodegenerative diseases [23], these cells may play a pivotal role in the development and regeneration of the central nervous system (CNS) via the synthesis and release of trophic factors, cytokines, and molecules from the extracellular matrix [24,25,26,27]. Tumor necrosis factor alpha (TNF-alpha) is a cytokine produced and released by activated microglia [28] that regulates the expression of neurotrophins and their receptors in glial cells [29] and monocytes [30], modulates neurite outgrowth [31,32,33], and acts as a neuroprotective agent in models of retinal ischemia [34]. Moreover, the endogenous production of TNF-alpha by astrocytes has also been associated with the production of nerve growth factor (NGF) and glial cell-derived neurotrophic factor (GDNF) [35]. Interestingly, TNF-alpha mediates the mitogenic activity of LGF in the liver [36]. Additionally, a single injection of LGF was found to be able to induce a transient increase of TNF-alpha protein expression and immunoreactivity in the lesioned striatum of hemiparkinsonian rats [19], so this cytokine could presumably be the molecular effector of LGF in our experimental model of PD.

Based on these studies, we hypothesize that the neuroprotective action of LGF on DA neurons observed in vivo is mediated by glial cells through the production of TNF-alpha. In order to prove this hypothesis, we used cultures of dopaminergic neurons and cultures of glia obtained from E14 (embryos from Sprague Dawley rats of 14 days) rat mesencephalon. The stimulation of glial cultures with LGF promoted the activation of the extracellular signal-regulated kinases 1/2 (ERK1/ERK2) and transcription factor CREB in a small population of microglia cells. In addition, LGF increased the expression of TNF-alpha in these cultures. The treatment of mesencephalic neurons with a glial-conditioned medium obtained from glial cell cultures previously treated with LGF (GCM-LGF) prevented the death of DA neurons induced by 6-hydroxy-dopamine (6-OHDA). This effect was not observed in the presence of a TNF-alpha activity blocking antibody. Altogether, these findings strongly suggest the involvement of microglia and TNF-alpha in the neuroprotective action of GCM-LGF on DA neurons observed in vitro.

## 2. Materials and Methods

### 2.1. LGF Purification

LGF was purified from the serum of 5-week bile duct-ligated Wistar rats following a previously reported procedure [10]. LGF was quantitated by HPLC [37], and samples with the highest serum LGF concentrations were selected to proceed with the purification process, which involved three chromatography steps employing Sephadex G-150, DEAE-cellulose, and hydroxyapatite. Purity, that is, the absence of other growth factors and/or contaminants in the LGF preparation, was also assessed according to standard criteria [10,11,12,38]. All LGF preparations showed a single band in sodium dodecyl sulfate polyacrylamide gel electrophoresis (SDS-PAGE). LGF preparations were lyophilized and kept at 4 °C until use, at which time aliquots were dissolved in saline prior to being used for the treatment of the cultures.

### 2.2. Ethics Statement

The Ethics Committee of the Hospital Ramón y Cajal, Madrid (animal facilities ES280790002001) approved all the protocols related to the use of laboratory animals. All procedures associated with animal experiments were in accordance with Spanish legislation (RD 53/2013) and the European Union Council Directive (2010/63/EU).

### 2.3. Midbrain Primary Cultures of E14 Rat Embryos

Embryos from Sprague Dawley rats of 14 days (E14), corresponding to a crown-rump length of 10–12 mm and a state of optimal embryo development, were used both to obtain cell cultures of mesencephalic glia and as cell cultures enriched in dopaminergic neurons. The dissection of the mesencephalon was performed in Leibovitz’s L-15 medium, as described in [39], and then the tissue was incubated for 15 min with Hank´s medium without calcium and magnesium, along with trypsin at 37 °C to promote the disaggregation of the cells. To inactivate and remove trypsin, the medium was removed and washed again with complete medium (CM) (modified Eagle’s medium with 4.5 g/l-glucose, 15% fetal bovine serum (FBS), 4 mM glutamine, 1 mM pyruvate, and 100 μg/mL of streptomycin), and then they were centrifuged at 400xG removing the supernatant. The cells were switched to a cryotube with CM. Once disrupted, we evaluated the number of live and dead cells by the method of Trypan blue exclusion in a Neubauer chamber. The cells were grown in CM at a density of 50,000 cells/cm^2^/(flask 75 cm2) for glia-enriched cultures at a density of 40,000 cells/cm^2^ (12 mm diameter glass coverslip) in 24-well plates for neuron-enriched cultures.

#### 2.3.1. Cultures Enriched in Neurons

The cultures enriched in mesencephalic neurons were performed as described in [40,41] with slight modifications. Briefly, the cells were kept in the incubator in a moist chamber at 37 °C in a 5% CO_2_ atmosphere. At 24 h after the seeding, CM was replaced with a serum-free defined medium (DM) consisting of Eagle’s minimal essential medium (EMEM) and a Ham F12 nutrient mixture (1:1) supplemented with 6 mg/mL of D-glucose, 25 g/mL of insulin, 100 mg/mL of human transferrin, 60 mM putrescine, 30 nM sodium selenite, and 20 nM progesterone. The cultures were kept 7 days in vitro. This serum-free medium maintained the population of dopaminergic cells by limiting the growth of non-neuronal cells.

#### 2.3.2. Glial Cultures

**Figure 1:**
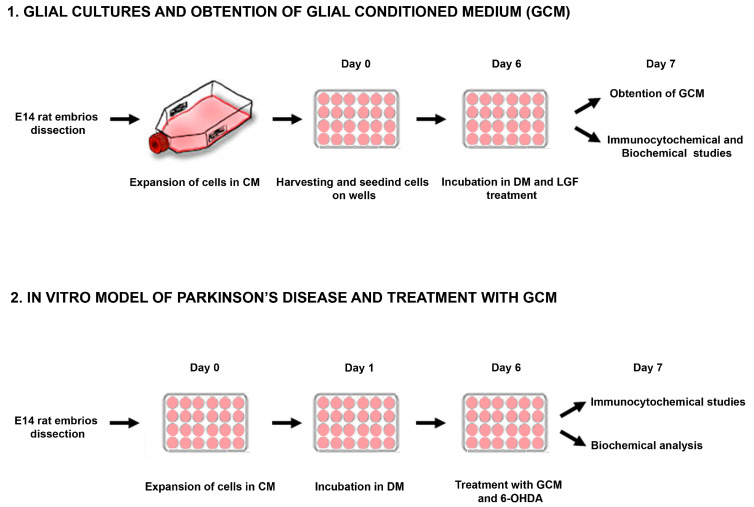
Experimental overview. Representative scheme showing the methodological procedure followed for obtention and treatment of glial cultures and mesencephalic neurons from E14 (embryos from Sprague Dawley rats of 14 days) rat mesencephalon. CM: complete medium; DM: defined medium; 6-OHDA: 6-hydroxy-dopamine.

Cells obtained from the midbrain of embryos (E14) were kept for 3–6 passages in CM, which promoted the growth of the glial population of the culture. Every 5–6 days, the medium was replaced. To obtain the GCM, the whole medium was discarded, and cells were washed 3 times with medium Leibovitz’s L15 to remove the rest of the serum, as described in [41] (Figure 1). Next, the cells were maintained in DM EF12 without serum for 24 h. Then, the medium was collected and frozen at −80 °C.

#### 2.3.3. Treatments

To cause the selective death of dopaminergic neurons, primary cultures of mesencephalic neurons were treated for 24 h with 40 µM of 6-OHDA, a neurotoxin that causes selective death of dopaminergic neurons in the SN [42]. Together with 6-OHDA, cultured neurons were treated with DM or conditioned medium from glial cultures treated with 100 pg LGF (GCM-LGF). 

In parallel, a group of dopaminergic neurons received vehicle in combination with DM, GCM, or GCM-LGF (Figure 1). Furthermore, together with 6-OHDA, primary cultures of mesencephalic neurons were treated with DM, GCM, or GCM-LGF in the presence or absence of an anti-mouse TNF-alpha blocking antibody (1 µg/mL) in order to assess the effect of the neutralization of the putative TNF-alpha bioactivity from the GCM-LGF.

### 2.4. Lactate Dehydrogenase Activity (LDH)

The rupture of the plasma membrane of cells induces the extracellular release of lactate dehydrogenase (LDH), which is a parameter indicative of cell death by necrosis. In neurons and glial mesencephalic cultures, the determination of LDH activity was done using a cytotoxicity detection kit (LDH) (Roche), evaluating the activity by spectrophotometry in the SPECTRAfluor (TECAN) at λ = 340 nm.

### 2.5. Immunocytochemical Characterization

The characterization of different cell phenotypes present in the cultures was made by immunocytochemical techniques. To facilitate handling, the cells were fixed for 10 min with paraformaldehyde (PFA) at 4% at pH 7.4, and then the coverslips were washed three times with phosphate buffered saline (PBS). Permeabilization was used 95/5 ethanol/acetic acid at −20 °C for 20 min, except in the case of phospho-ERK, and phospho-CREB was used 0.05% Triton in PBS for 5 min at 4 °C. When the antigen was a surface antigen like in the case of O4, this step was not done, performing a complete immunocytochemistry for the surface antibody. The coverslips were placed in a moist chamber and before to blockade them with PBS and normal goat serum (NGS) (5%) for half an hour at room temperature. Later, the blockade was removed, and each coverslip was incubated with 50 ul of the concentration described for the corresponding primary antibody overnight at 4 °C.

#### 2.5.1. Antibodies

The primary monoclonal antibodies applied were: anti-mouse tyrosine hydroxylase (TH, 1:500, Chemicon International Inc) and anti-mouse ERK1/2 di (^Thr183/Tyr185 P^) (1:100, Sigma). The polyclonal antibodies used were: anti-β-tubulin isotype III of rabbit (1:1000, Covance), anti-glial acidic protein (GFAP) of rabbit (1:500 DakoCytomation), and anti phospho-CREB of rabbit (1:500, Upstate). Moreover, an anti-mouse TNF-alpha blocking antibody (1 µg/mL, R&D Systems) was used in order to neutralize TNF-alpha bioactivity in culture.

For an immunofluorescent assay, the secondary antibodies used were anti-rabbit IgG from goat conjugated to Alexa Fluor-488 antibody and anti-mouse IgG goat conjugated to Alexa Fluor-568 (both 1:400, Molecular Probes, Eugene, OR).

#### 2.5.2. Immunofluorescence

In the detection of neuronal marker β-tubulin III, TH, as well as for the visualization of astrocytes by GFAP, immunocytochemical techniques were applied to those secondary antibodies conjugated to fluorochromes. In addition, for the observation of cell nuclei, sections were mounted in an aqueous medium containing bisbenzimide (Hoechst 33342, Sigma).

#### 2.5.3. Immunoperoxidase

For the detection of isolectin B4 (IB4), which is used to express microglia and/or macrophages in coverslips after being blocked, was incubated with isolectin IB4 conjugated with peroxidase (1:20, Sigma) for 2 h. The reaction product was detected with 3,3′-Diaminobenzidine (DAB) and a substrate-chromogen system.

For the morphometric analysis, we used a fluorescence microscope coupled to a computerized stereological analysis system (CAST Grid, Olympus, Ballerup, Denmark). It defined the area of each coverslip and quantified the number of cells with a specific staining using a 20× objective. The results were expressed as a percentage of immunopositive cells/mm^2^ compared to total cells/mm^2^, and represent the mean ± SEM of 6–10 coverslips from 3 individual experiments.

### 2.6. Biochemical Study

#### 2.6.1. Collection and Homogenization of the Cells for Protein Extraction

In glial cultures, once treated with vehicle or LGF, the medium was removed and washed with sterile PBS to lift the cells that were centrifuged for 5 min at 4 °C at 400 *g*. Subsequently, the supernatant was removed, and a 20 mM Tris–HCl lysis buffer (pH 7.5) was added; this contained 140 mM KCl, 5 mM Mg (CH3COO) 2, 1 mM dithiothreitol (DTT), 1 mM ethylenediaminetetraacetic acid (EDTA), 2 mM ethylene glycol-bis(β-aminoethyl ether)-N,N,N,N-tetraacetic acid (EGTA), specific inhibitors of proteases (10 μg/mL pepstatin A, 10 μg/mL leupeptin and, 10 μg antipain), and specific inhibitors of phosphatases (20 mM β-glycerophosphate, 2 mM benzamidine, 20 mM sodium molybdate, and 200 mM sodium orthovanadate). The cell lysate was centrifuged for 20 min at 4 °C 11,000 *g* to collect the supernatant.

Protein concentration was determined by the bicinchoninic acid assay (BCA) method (Protein Assay, ThermoScientific) using as a standard bovine serum albumin. The procedures were performed at 4 °C, and samples were kept at −80 °C until use.

#### 2.6.2. Western Blot

Aliquots of 30 µg of protein were separated by polyacrylamide gel electrophoresis (SDS-PAGE). To do this, we used a vertical electrophoresis system analytical Bio-Rad protein (TetraCell). The polyacrylamide gels were 1 mm thick in the presence of sodium dodecyl-(SDS) at 0.1% (electrophoresis dissociative). We used NNN’-N’-tetramethylene diamine (TEMED) 0.86% and ammonium persulfate (2.14 ug/uL) as polymerisation agents. The samples were prepared in a loading buffer (180 mm Tris–HCl, pH 6.8 with 9% SDS, 6% β-mercaptoethanol, 15% glycerol, and 0.025% bromophenol blue) and resolved at 200 V for 1 h.

The buffer used to develop the electrophoresis was composed of Tris–glycine at pH 8.3 (250 mM Tris and 192 mM glycine) and 0.1% SDS. The proteins separated by electrophoresis were transferred to nitrocellulose membranes using a wet transfer system. For SDS electrophoresis, the transfer system used was a continuous system containing Tris–glycine/methanol (Tris 25 mM, 192 mm glycine, and 20% methanol), and the electrophoresis was carried out for 1 h at a constant voltage of 100 V and 4 °C.

Membranes were soaked in a blocking solution (0.1 M PBS and 5% dry skimmed milk, pH 7.4) and incubated with the following primary antibodies diluted in 0.1 M PBS and 1% dry skimmed milk at pH 7.4: For the immunodetection of markers of microglia, we used a mouse monoclonal anti-ionized calcium binding adaptor molecule 1 (Iba1) (17 kDa) (1:500, Millipore), and for astrocytes, we used anti-GFAP (50 kDa) (1:5000). Additionally tested were ERK1/2 di (Thr183yTyr185 P)/(42/44 kDa) (1:5000), ERK1/2 (42/44 kDa) (1:10,000), TH (52 kDa) (1:5000), P-CREB (43 kDa) (1:1000), and TNF-α (17 kDa) goat polyclonal (1:400, Santa Cruz Biotech). After extensive washing in 0.05% PBS–Tween, membranes were incubated with secondary antibodies directed against the species in which the primary antibody was obtained, and then they were conjugated with peroxidase (1:2000, Amersham Pharmacia Biotech).

The membranes were developed with enhanced chemiluminescence Western blotting, following the manufacturer’s instructions (Amersham), and then they were exposed to hyperfilm. Membranes were also immunolabeled for loading control using mouse anti-β-actin (1:5000; Sigma Aldrich) and phosphatase-conjugated anti-mouse IgG alkaline (1:3000, Sigma Aldrich). Then, the membranes were developed using an alkaline phosphatase reagent. The density of the stained bands was scanned and quantified with the Image QuantTL software package, and the data were normalized with respect to β-actin levels.

### 2.7. Glutathione Determination

The method for the determination of glutathione was HPLC. The mobile phases used were A: sodium acetate buffer, 0.5 M pH 6.8, filtered using a 0.45 μm filter pore; and B: acetonitrile. The column was a BECKMAN Ultrasphere-Octadecyl-silica (ODS) reverse phase C18 tempered to 35.5 °C with a particle size of 5 μm. The reagent for derivatization was OPA (o-phthalaldehyde), and reading was done with a fluorometer. The wavelengths were set at 365 nm for excitation and 455 nm for emission. The glutathione concentration of the standard was 1 μM, 20 μL of which were injected for analysis at a flow rate of 1 mL/min.

### 2.8. Statistical Analysis

Results are expressed as mean ± SEM from 6 to 10 coverslips from 3 independent experiments. For Western blot and biochemical analysis, experiments represent the mean ± SEM from 3 to 9 independent experiments. Statistical analysis was performed with the GraphPad Prism software (La Jolla, CA, USA). Before analysis, the Shapiro–Wilk test was used to test normality. For parametric data, a Student´s t-test or one-way ANOVA followed by the Newman–Keuls multiple comparison test were performed. Differences were considered significant when *p* ≤ 0.05.

## 3. Results

### 3.1. Characterization of Mesencephalic Glial Cultures

**Figure 2:**
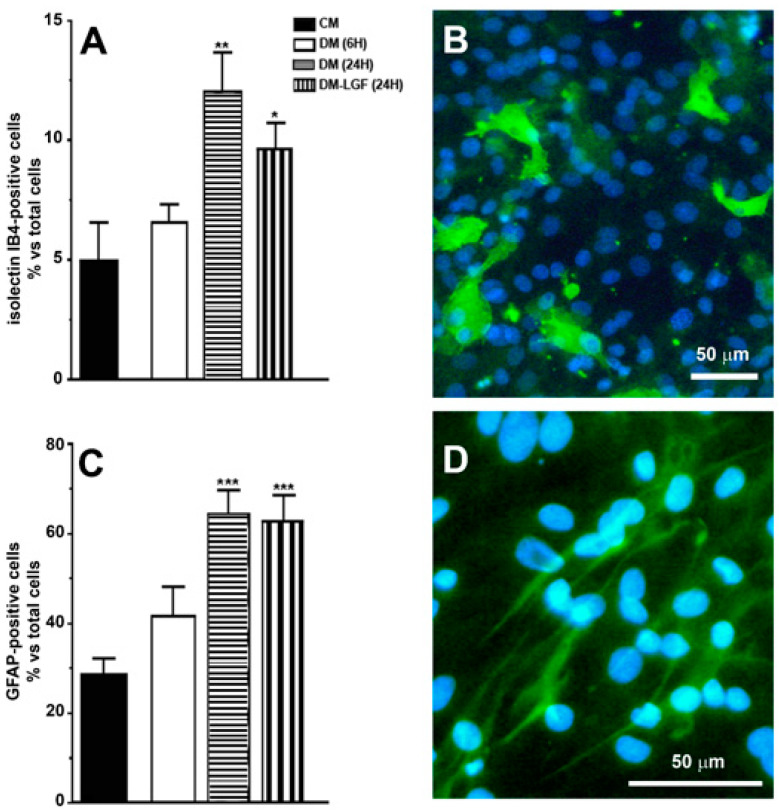
Immunocytochemical characterization of cultures of mesencephalic glia. (**A**,**C**) show the percentage of immunopositive cells for isolectin IB4 and anti-glial acidic protein (GFAP), specific markers for microglia and astrocytes, respectively. (**B**) shows the immunodetection for isolectin IB4 (green), and (**D**) for GFAP (green). Cell nuclei were stained with Hoechst (blue). In (**A**,**B**), the results represent the mean ± SEM of 8 coverslips from 2 independent experiments. CM (complete medium), DM (defined medium), DM-LGF (defined medium with liver growth factor). * *p* ≤ 0.05, ** *p* ≤ 0.01, *** *p* ≤ 0.001 vs. CM. Scale bar 50 µm.

Our previous studies suggested that microglia and/or astrocytes are potential mediators of the neuroprotective activity of LGF observed in vivo [16,17,19]. In this study, we used mixed glial cultures obtained from mesencephalon of E14 rat embryos. In the presence of 15% FBS (CM), 28% of cells in these cultures were GFAP-positive astrocytes, and about 5% were microglia isolectin IB4-positive cells (Figure 2A–D). To analyze the potential effects of LGF, glial cultures were maintained for 6 or 24 h in a defined medium devoid of FBS (DM). As shown in Figure 2, the lack of FBS for 24 h significantly increased GFAP- and isolectin IB4-positive cells, as compared with those cultures that were maintained in CM. Neither GFAP- nor isolectin-positive cells were affected after 6 h of FBS depletion (*p* = 0.111 and *p* = 0.407, respectively) (Figure 2A,B).

### 3.2. LGF Stimulates Microglia in Mesencephalic Glial Cultures

**Figure 3:**
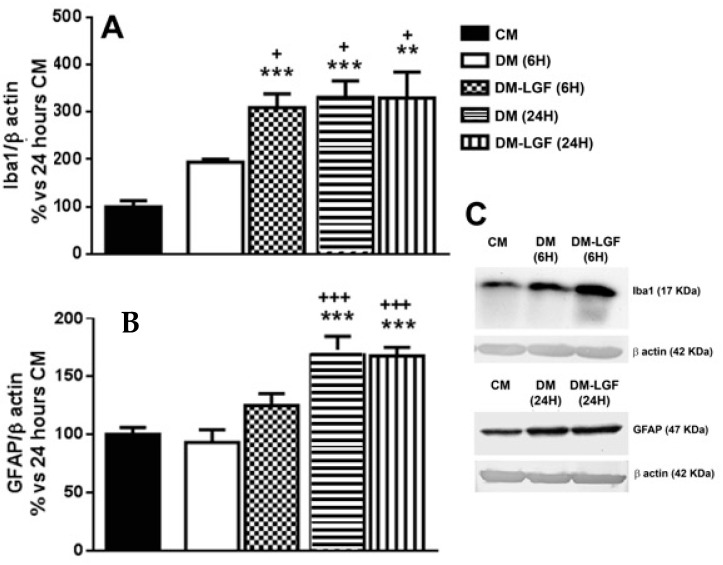
Liver growth factor (LGF) modulated Iba1 protein expression in cultured mesencephalic glia. (**A**) shows how 100 pg/mL of LGF treatment up-regulated Iba1 protein expression after 6 h (dots bar) and 24 h of treatment (vertical black lines bar), as compared to cultures maintained in complete medium (CM) (black bar) and defined medium (DM) (white bar) for 6 h. Iba1 protein levels were also increased in cultures maintained in DM for 24 h (**A**) (horizontal black lines bar). GFAP expression was also up-regulated after 24 h of DM (**B**) (horizontal black lines bar) and DM-LGF (**B**) (vertical black lines bar). (**C**) shows representative bands of Iba1 and GFAP. Results represent the mean ± SEM of 4 (**A**) and 5 (**B**) independent experiments. ** *p* ≤ 0.01 and *** *p* ≤ 0.001 vs. CM, + *p* ≤ 0.05, +++ *p* ≤ 0.001 vs. 6 h DM (ANOVA and Newman–Keuls).

Microglia are potential mediators of the neuroprotective activity of LGF observed in vivo [16,17,19]. As mentioned above, microglia represented the 6.55 ± 0.75% of total cells in cultures maintained in DM for 6 h (Figure 2A). LGF treatment for 6 h did not significantly modify the percentage of isolectin IB4-positive cells (7.77 ± 1.64%, *p* = 0.407), but it significantly up-regulated Iba1 protein expression, which is a feature of microglia activation (Figure 3A). Iba1 protein levels were also increased after 24 h of LGF treatment, but at this experimental time, no differences were observed in comparison with those cultures maintained in DM (*p* = 0.972) (Figure 3A). Similarly, LGF treatment was unable to modulate the percentage of microglia isolectin IB4-positive cells that, at this experimental time, represented a 12 ± 1.63% of total cells (Figure 2A). Altogether, these results strongly suggested that the LGF-induced activation of microglia was an early and maintained effect in our cultures.

We also analyzed the potential ability of LGF to stimulate GFAP-positive astrocytes. This population of cells was significantly increased from 28% to 64% of the total cells in those cultures that were maintained in DM or DM-LGF for 24 h (Figure 2A). At this experimental time, GFAP protein expression was also increased by 1.7-fold in DM- and DM-LGF-treated cultures (Figure 3B,C).

### 3.3. LGF Promotes the Activation of ERK1/2, CREB in Mesencephalic Glial Cultures

**Figure 4:**
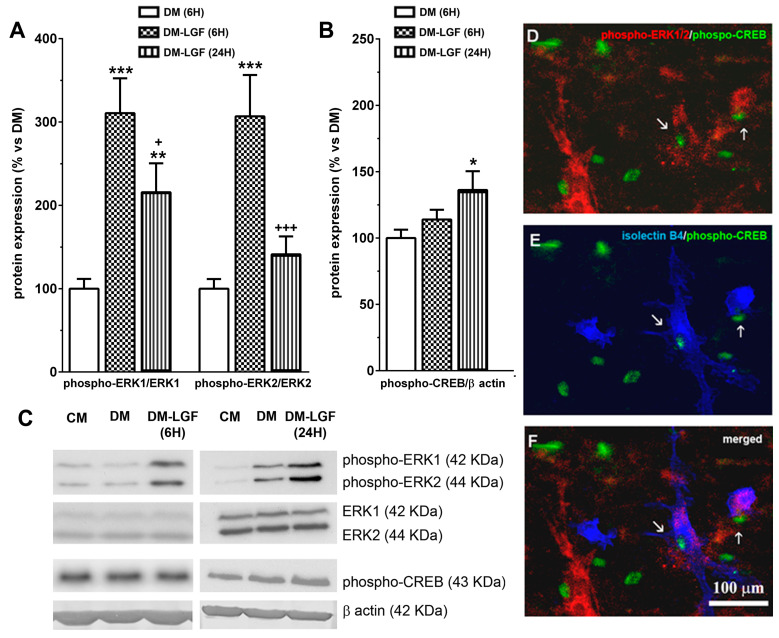
Effects of LGF on ERK1/2 and CREB phosphorylation in cultured mesencephalic glia. (**A**) shows the effect of LGF on phospho-ERK1/ERK1 and phospho-ERK2/ERK2 ratios in mesencephalic glia maintained in a DM (white bar) (**A**) and in a defined medium with LGF (DM and LGF) for 6 (dots bar) or 24 h (black bar). Note how LGF increased the phospho-ERK1/ERK1 ratio at both experimental times. (**B**) shows the effect of LGF treatment in phospho-CREB protein expression in DM (white bar) and in a defined medium with LGF (DM and LGF) for 6 (dots bar) or 24 h (vertical black lines bar). Note how LGF treatment for 24 h significantly increased phospho-CREB levels. (**C**) shows representative bands of phospho-ERK1/2, ERK1/2, phospho-CREB, and β-actin. Note how ERK1/2 protein levels are unchanged under all experimental conditions. (**D**–**F**) show how some phospho-CREB-positive cells—(**D**–**F**) in green—were also immunopositive for phospho-ERK1/2 (**D**) (red) and isolectin IB4 (**E**) (blue), as well as how a few isolectin IB4-positive cells were phospho-ERK1/2- and phospho-CREB-positive too (**F**). Results in (**A**,**B**) represent the mean ± SEM of 5–9 independent experiments. * *p* ≤ 0.05, ** *p* ≤ 0.01, *** *p* ≤ 0.001 vs. DM, + *p* ≤ 0.05, +++ *p* ≤ 0.001 vs. 6 h DM and LGF (ANOVA and Newman–Keuls). In (**C**), lane 1 and 4: CM; lane 2 and 5: DM; lane 3: DM and LGF (6 h); and lane 6: DM and LGF (24 h). Scale bar 100 µm.

LGF in vivo administration promotes the activation of MAPKs ERK1/2 and CREB [19]. To determine the possible involvement of glial cells in these LGF actions, we analyzed the expression of phospho-ERK1/2 and phospho-CREB in mesencephalic glial cultures treated for 6 or 24 h with a defined medium devoid of FBS in the absence (DM), or presence of 100 pg/mL of LGF (DM-LGF). After 6 h of LGF treatment, phospho-ERK1 and phospho-ERK2 levels were increased, as compared to those cultures that were kept in DM (Figure 4A,C). Increased phospho-ERK1 levels were also observed in glial cultures treated for 24 h with the factor (Figure 4A,C). Since LGF did not alter the expression of ERK1/2 at any experimental time analyzed in this study (Figure 4C), our results indicated that the factor was able to stimulate this signaling pathway in glial cells.

The activation of ERK1/2 could promote the phosphorylation of the transcription factor CREB. LGF also up-regulated CREB phosphorylation by 1.4-fold, but this effect was only observed after 24 h of treatment (Figure 4B,C). Immunocytochemical studies showed that a small population of cells were double immunostained for phospho-ERK1/2 and phospho-CREB in LGF-treated cultures (Figure 4D). Additionally, these cells showed cytochemical reactivity for isolectin-IB4 (Figure 4E,F), suggesting that microglia could have been a target for LGF in these cultures.

### 3.4. LGF Effects on TNF-Alpha Expression and Glutathione Concentration in Mesencephalic Glial Cultures

**Figure 5:**
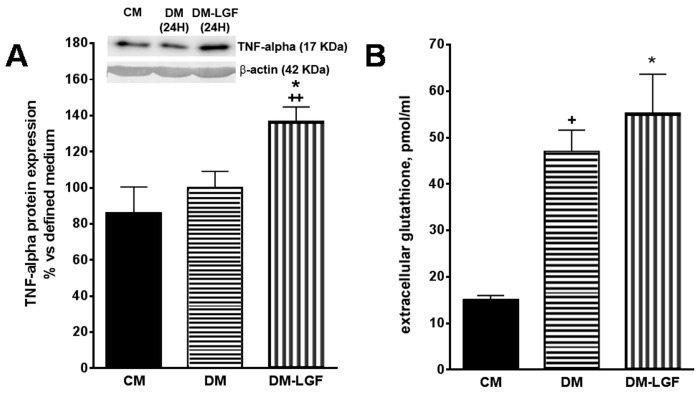
Effects of LGF on tumor necrosis factor alpha (TNF-alpha) protein expression and extracellular glutathione modulation in mesencephalic glial cultures. (**A**) shows how 100 pg of LGF increased TNF-alpha protein expression in mesencephalic glial cultures, as compared with those cultures maintained in a defined medium for 24 h (DM) or in a complete medium containing 15% FBS (CM). (**B**) shows how the depletion of FBS increased the extracellular concentration of glutathione in glial cultures, an effect that was not modulated by LGF treatment. The results represent the mean ± SEM of 4–5 independent experiments. * *p* ≤ 0.05 vs. DM, ++ *p* ≤ 0.01 vs. CM in A, * *p* ≤ 0.05 and + *p* ≤ 0.05 vs. CM in B (ANOVA and Newman–Keuls). In A lane 1: CM; lane 2: DM (24 h); lane 3: DM-LGF (24 h).

The stimulation of ERK1/2 and CREB in glia could promote the synthesis and release of neuroprotective factors of a dopaminergic system. Recent work has suggested that the cytokine TNF-alpha may play an important role in neuroregeneration [31,32,33,34]. Given that TNF-alpha is the molecular mediator of the regenerative action of LGF in liver [36] and that this cytokine is expressed by glial cells [28], we analyzed the effect of LGF on the expression of TNF-alpha in mesencephalic glia cultures by Western blot techniques. Figure 5A shows how a 100 pg/mL LGF treatment for 24 h increased the intracellular expression of TNF-alpha by 1.4-fold.

Other authors have shown that glutathione released by glial cells protects dopamine neurons from damage caused by L-DOPA [41,43,44,45]. LGF treatment increases glutathione plasmatic levels in hypertensive rats [46], so this antioxidant may be one of the mediators of the neuroprotective action of GCM-LGF. Using HPLC techniques, we determined the extracellular concentration of glutathione in cultured glial cells treated and not treated with LGF. Compared with the glial cell cultures maintained in the presence of FBS, the extracellular concentration of glutathione increased significantly in both experimental situations (Figure 5B).

### 3.5. Development of an In Vitro Model of PD

**Figure 6:**
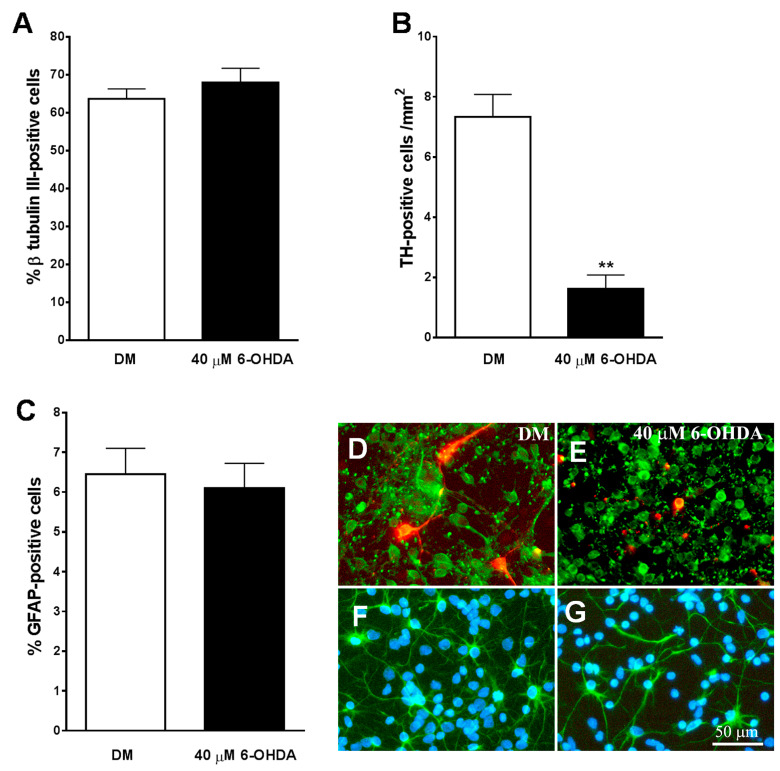
Effect of 6-OHDA on cultures of mesencephalic neurons. (**A**–**C**) show the effect of 40 µM of 6-OHDA on total population of neurons (**A**), tyrosine hydroxylase (TH)-positive dopaminergic neurons (**B**), and astrocytes (**C**). Note how the neurotoxin specifically decreases the population of TH-positive neurons (**B**). (**E**) shows how in the presence of 6-OHDA, both the total population of neurons β-tubulin III-positive ((**D**,**E**) in green) and the TH-positive dopaminergic neurons ((**D**,**E**) in red) lost their neuritic extensions and had a smaller size of their cell bodies, as compared with those cells grown in DM (**D**). (**F**,**G**) show immunostaining for GFAP-positive astrocytes (green) and cell nuclei stained with Hoechst (blue). Note how 6-OHDA (**G**) did not affect to this cell population. The results in (**A**–**C**) represent the mean ± SEM of 8 coverslips from 3 independent experiments. ** *p* ≤ 0.01 vs. DM (t-test). Scale bar 50 µm.

We used cultures of neurons obtained from the midbrain of E14 to be treated with the specific neurotoxins of 6-OHDA catecholaminergic neurons as an experimental model of PD in vitro. An immunocytochemical analysis of these cultures revealed that approximately 65% of the cells expressed the neuronal marker β-tubulin III (Figure 6A,D), and only 6% of the total population of cells expressed the astroglial marker GFAP (Figure 6C,F). Additionally, 7.3 ± 0.75 cells/mm^2^ expressed the specific marker for dopaminergic neurons TH in these cultures (Figure 6B,D) and represented a 0.6 ± 0.05 % of β-tubulin III-positive neurons in these cultures.

As shown in Figure 6B, the number of TH-positive cells/mm^2^ decreased significantly in midbrain cultures treated for 24 h with 40 µM of 6-OHDA. In addition, the remaining TH-positive neurons had a lack of the neuritic extensions (Figure 6E), indicating that the dopaminergic neurons of the cultures were heavily damaged after treatment with the neurotoxin. The percentage of β-tubulin III-positive cells was not reduced by the addition of 40 µM of 6-OHDA to the cultures (*p* = 0.354) (Figure 6A). However, the neurotoxin visibly affected their morphology, showing smaller cell bodies and reducing neuritic extensions than untreated neurons (Figure 6D,E).

Treatment with 6-OHDA 40 µM did not affect the percentage (*p* = 0.707) and morphology of GFAP-positive cells in the culture (Figure 6C,F,G). On the other hand, a lower dose of 6-OHDA (20 µM) did not significantly reduce the percentage of total and TH-positive neurons in the cultures (*p* = 0.399).

**Figure 7:**
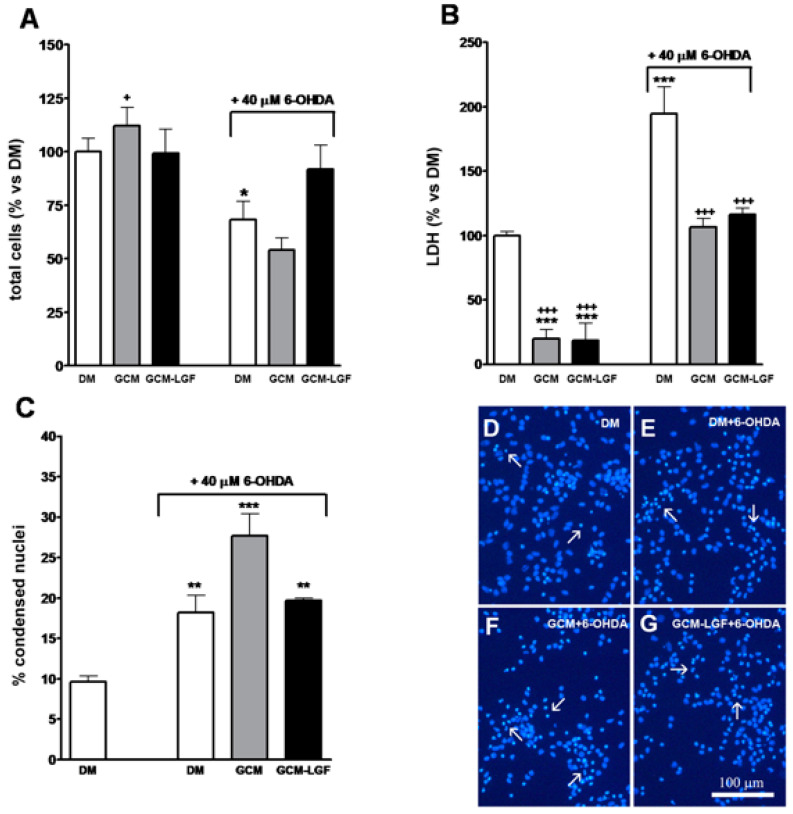
Effects of glial-conditioned medium from LGF-treated glial cultures (GCM-LGF) on lactate dehydrogenase (LDH) activity and neuronal degeneration in mesencephalic neurons damaged with 6-OHDA. (**A**–**C**) show the effect of GCM and GCM-LGF on total cells (**A**), extracellular lactate dehydrogenase (LDH) (**B**), and percentage of apoptotic nuclei (**C**) on midbrain neuronal cultures treated with 40 µM of 6-OHDA. Note how GCM-LGF prevented the decrease in total number of cells caused by the neurotoxin (**A**) and how GCM reduced cellular death by necrosis in the cultures (**B**). Panels (**D**–**G**) show how degenerated cell nuclei stained with Hoechst that had condensed chromatin (white arrows) were present in neuronal cultures treated with 6-OHDA in the absence (**E**) and presence of GCM (**F**) or GCM-LGF (**G**), as well as how GCM increased the percentage of these nuclei showing condensed chromatin (**C**) or lacking for condensed chromatin (**F**). Results in (**A**–**C**) represent the mean ± SEM of 6–9 coverslips from 3 independent experiments. * *p* ≤ 0.05, ** *p* ≤ 0.01, *** *p* ≤ 0.001 vs. DM, + *p* ≤ 0.05, +++ *p* ≤ 0.001 vs. 40 uM 6-OHDA (ANOVA and Newman–Keuls). DM, GCM, and GCM-LGF. Scale bar 100 μm.

In addition, treatment with 40 µM of 6-OHDA for 24 h decreased total number of cells in the cultures (Figure 7A) and significantly increased extracellular concentration of LDH (Figure 7B), and the number of nuclei showing condensed chromatin, as analyzed by Hoechst staining (Figure 7C,E). These results indicated that, under our experimental conditions, 40 µM of 6-OHDA promoted necrosis and neuronal degeneration in midbrain cultures.

### 3.6. GCM Effects on Mesencephalic Neuronal Cultures Damaged by 6-OHDA

Glial stimulation with LGF could promote the synthesis and/or release of factors capable of protecting mesencephalic neurons from damage caused by 6-OHDA. To examine this possibility, mesencephalic neuronal cultures were treated with a conditioned medium obtained from glial cultures not treated with LGF (GCM) or treated with the factor (GCM-LGF). Twenty-four hours later, cultures were fixed, and their contents of total neurons and TH-positive cells were analyzed.

**Figure 8:**
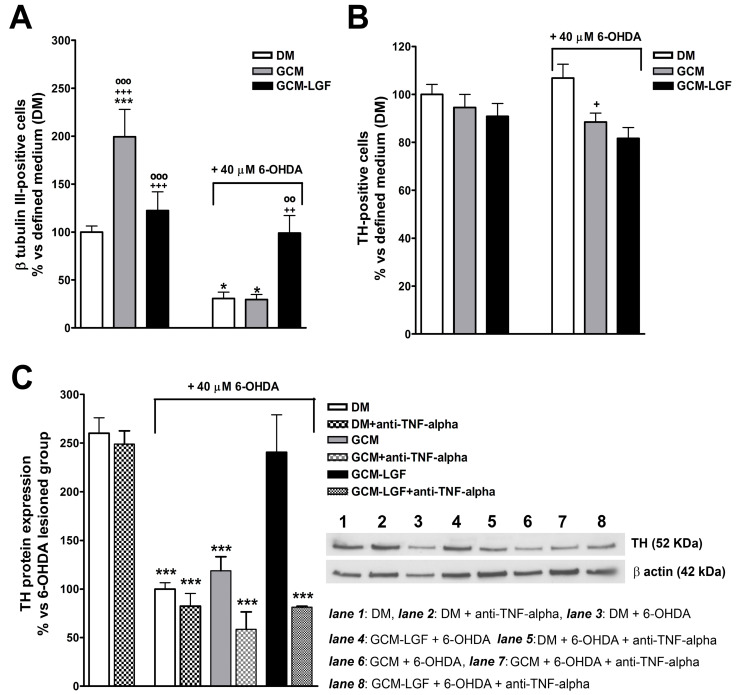
Effects of glial-conditioned medium obtained from LGF-treated mesencephalic glia on dopaminergic neurons. By using immunocytochemistry, we analyzed the effect of GCM (grey bars) and glial-conditioned medium obtained from 100 pg/mL GCM-LGF (black bars) on the population of TH-positive (**A**) and β-tubulin III-positive cells (**B**) in 40 µM of 6-OHDA-treated mesencephalic neuronal cultures. Note how GCM-LGF prevented the decrease in the percentage of TH-positive cells promoted by the neurotoxin (**A**). The results represent the mean ± SEM of 7–10 coverslips from 3 independent experiments. * *p* ≤ 0.05, *** *p* ≤ 0.001 vs. DM (white bars), + *p* ≤ 0.05, ++ *p* ≤ 0.01, +++ *p* ≤ 0.001 vs. DM and 40 µM of 6-OHDA., **◦◦** *p* ≤ 0.01, **◦◦◦** *p* ≤ 0.001 vs. GCM and 40 µM of 6-OHDA. (**C**) shows the Western blot analysis of TH expression in mesencephalic cultures. Note how GCM-LGF preserved TH reduction, as promoted by 40 µM of 6-OHDA (black bar), as well as how this neuroprotective effect was not observed when cultures were treated with GCM-LGF in the presence of 1 µg/mL anti-TNF-alpha blocking antibody (last dot bar). In (**C**), lane 1: DM; lane 2: DM and anti-TNF-alpha; lane 3: DM and 6-OHDA; lane 4: DM, 6-OHDA and anti-TNF-alpha; lane 5: GCM and 6-OHDA; lane 6: GCM, 6-OHDA and anti-TNF-alpha; lane 7: GCM-LGF and 6-OHDA; and lane 8: GCM-LGF, 6-OHDA and anti-TNF-alpha. The results represent the mean ± SEM of 3 independent experiments. *** *p* ≤ 0.001 vs. DM (ANOVA and Newman–Keuls).

The treatment of mesencephalic neurons with GCM significantly increased the percentage of TH-positive cells (Figure 8A). Compared with cultures maintained in a DM, treatment with GCM-LGF did not affect the percentage of TH-positive cells (*p* = 0.303) (Figure 8A).

GCM protects dopaminergic neurons from damage caused by several neurotoxins [41,47]. In the used in vitro model of PD, GCM did not prevent the decrease in the number of TH-positive cells (Figure 8A). However, GCM-LGF protected TH-positive neurons from the harmful effects of the neurotoxin, avoiding the decrease in their number (Figure 8A—black bar on the right). Western blot analysis also showed how GCM-LGF prevented the reduction of TH protein expression promoted by 6-OHDA, compared to those cultures treated with GCM that did not show this effect (Figure 8C—black bar and grey bar, respectively).

As shown in Section 3.4, LGF up-regulated TNF-alpha protein levels in glial cultures. To determine if TNF-alpha mediates LGF-induced neuroprotection, mesencephalic neurons were treated with GCM-LGF in the presence of 1 µg/mL of an anti-TNF-alpha blocking antibody. Under these experimental conditions, GCM-LGF was unable to preserve the reduction in TH protein expression promoted by 40 µM of 6-OHDA in these cultures (Figure 8C—last dot bar). These results strongly suggested that TNF-alpha could be a potential molecular effector of LGF in this in vitro model of PD.

Both treatments (GCM or GCM-LGF) slightly reduced the population of β-tubulin III-positive cells in 6-OHDA-treated neurons (Figure 8B). On the other hand, these treatments reduced the extracellular LDH content observed in mesencephalic neurons treated with 40 µM of 6-OHDA (Figure 7B). However, although GCM-LGF prevented the decrease in the total number of cells caused by 6-OHDA in neuronal cultures (Figure 7A), neither GCM nor GCM-LGF protected cells from death induced by the neurotoxin (Figure 7C,F,G).

**Figure 9:**
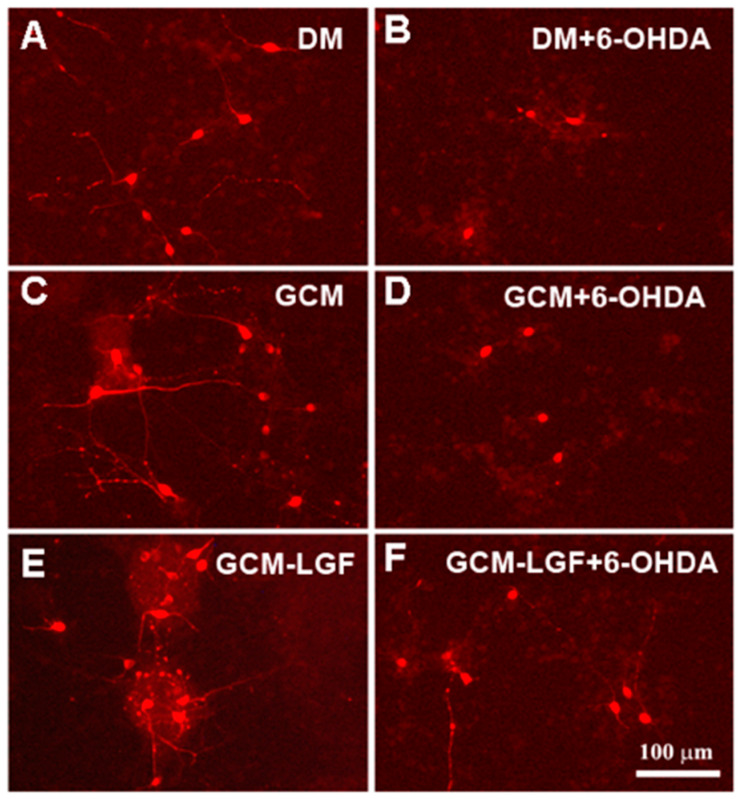
Effects of GCM-LGF on the morphology of midbrain dopaminergic neurons in culture. (**A**–**F**) show the effects of treatment with GCM and GCM-LGF on TH-positive midbrain neurons in culture. Note how GCM-LGF partially prevented the loss of neuritic extensions of TH-positive cells induced by 6-OHDA (**F**). Scale bar 100 µm.

GCM exerted a trophic effect on mesencephalic neurons because their cell bodies were more robust and presented longer neuritic extensions compared to the DM group (Figure 9A,C). GCM-LGF did not change the morphology of the TH-positive cells (Figure 9A,E), but it did protect them from part of the morphological damage caused by 6-OHDA compared to the effects observed in the GCM-treated cells (Figure 9F,D, respectively).

## 4. Discussion

In this article, we have shown the relevance of glial cells in the neuroprotective effects exerted by LGF on dopaminergic neurons in an experimental in vitro model of PD. The application of LGF to cultures of mesencephalic glia promoted the activation of MAPKs ERK1/2 and CREB phosphorylation in a small population of microglia. Additionally, LGF activated microglia and increases TNF-alpha intracellular levels in these cultures. In the presence of an anti-TNF-alpha blocking antibody, the GCM obtained from LGF-treated glial cultures lacked for neuroprotective activity. Consequently, our results suggested that this cytokine may be the molecular effector that mediates the above-mentioned neuroprotective effect of LGF.

Our previous results suggested that microglia and/or astrocytes could mediate the neuroprotective effect of LGF on dopaminergic neurons observed in vivo [16,17,19]. As shown here, LGF was unable to modulate the differentiation of mesencephalic glial cells in culture, but this factor stimulated those cultures by promoting the activation of MAPKs ERK1/2. Similar results have been obtained by other authors, suggesting how albumin (LGF is an albumin–bilirubin complex) activates the MAPKs ERK1/2 pathway in astrocytes and microglia [48]. In addition, our in vivo studies also showed increased ERK1/2 phosphorylation in the denervated striatum of 6-OHDA-lesioned rats treated with LGF [19]. ERK1/2 activation can promote the phosphorylation of CREB, a transcription factor that is involved in the synthesis of several factors that have neurotrophic activity [49]. In mixed cultures of mesencephalic glia, LGF stimulated the levels of phosphorylated CREB by 1.4-fold. In addition, the double immunolabeling of ERK1/2 and phospho-CREB were observed in a small population of isolectin IB4-positive cells, indicating that LGF was able to stimulate microglia in these cultures. According to present results, LGF up-regulated Iba1 protein expression and increased TNF-alpha protein levels. These are two features associated with the transient activation of microglia that were also observed in our in vivo studies [15,19].

Activated microglia have been associated with the pathogenesis of PD [23,45], but these cells may have a role in CNS regeneration [24,25,26,27]. In fact, recent work has shown that conditioned medium obtained from microglia protects neuronal death induced by 6-OHDA [47]. In this study, we examined the potential ability of GCM obtained from LGF-treated glial cultures to protect mesencephalic neurons from 6-OHDA neurotoxicity. The treatment of mesencephalic neuronal cultures with the neurotoxin affected cell viability by increasing LDH levels in the extracellular medium (death by necrosis) and the number of degenerated cells showing nuclei with condensed chromatin. In addition, the neurotoxin significantly reduced the number of TH-positive neurons in the cultures. Several studies have shown how GCM protects dopaminergic neurons from the L-DOPA, 1-methyl-4-phenylpyridinium (MPP+) and 6-OHDA toxicity [41,44,47]. However, in our in vitro model of PD, although GCM decreased extracellular LDH activity, this treatment was unable to preserve the harmful effects of 6-OHDA on TH-positive cells and increased cell death. By contrast, GCM-LGF prevented the loss of both TH-positive and total cells caused by 6-OHDA. These findings strongly suggested that the GCM-LGF contains factors with specific neuroprotective activity on TH-positive neurons.

Glial cells can produce and secrete pro-and anti-inflammatory molecules, antioxidants, and neurotrophic factors that could maintain the survival of dopaminergic neurons in culture [22,50]. The activation of astroglia [51] and microglia by several stimuli promotes the synthesis and secretion of various factors with neuroprotective activity [52,53,54]. Among them, the cytokine TNF-alpha seems to play an important role in the mitogenic cascade of LGF in the liver, as well as in LGF-induced neuroregeneration (reviewed by Gonzalo-Gobernado et al., 2014) [15]. Moreover, several studies have shown the involvement of TNF-alpha in neuronal survival [21,55,56] and neurites outgrowth [57]. As present results showed, in the presence of an anti-TNF-alpha blocking antibody, GCM-LGF was unable to preserve TH-positive neurons from 6-OHDA neurotoxicity, suggesting that TNF-alpha is a molecular effector responsible for the neuroprotective effect of GCM-LGF observed on dopaminergic neurons in culture. However, we cannot exclude the involvement of other factors with neuroprotective activity released by glial cells such as the neurotrophins GDNF and BDNF [53,54] or transforming growth factor beta 1 (TGFβ1), which is involved in the activation of microglia [58]. In addition, the neuroprotective properties of TNF-alpha are associated with its ability to induce BDNF expression in astrocytes [51]. In this respect, we should mention that LGF induced the synthesis of TGF-β1 in CCI4-cirrhotic rats [14] and up-regulated the expression of the neurotrophin receptor Trk B by 1.5-fold (*n* = 4, *p* ≤ 0.05) in cultures of mesencephalic glia. From our previous results, using experimental models of PD, we are not able to elucidate if the neuroprotective effects of LGF are related to specific mechanisms in order to cross the blood–brain barrier [17,18,19]. One mechanism could be the ability of LGF to bind to specific receptors found in the endothelium known as RAGE receptors (receptors for advanced glycation end products) [59]. Interestingly, advanced glycation end products (AGE) show analogous biochemical and structural properties to LGF, the structure of these products is mainly based on albumins bound to glucose with the ability to change albumin conformation [60]. Moreover, astrocytes and microglia express RAGE [61], and the activation of these receptors stimulates TNF-alpha release [62].

On the other hand, LGF increased the total glutathione levels in the plasma of hypertensive SHR rats [46]. Glutathione is an antioxidant that protects dopaminergic neurons from damage caused by various neurotoxins [63]. It can also be produced and released by glial cells [64]. Our results showed that in the absence of FBS, glial cells increased the secretion of glutathione into the extracellular medium, both in the absence and in the presence of LGF. Given that the GCM failed to protect dopamine neurons from 6-OHDA neurotoxicity, it seems reasonable to think that this antioxidant is not responsible for the above-mentioned neuroprotective effect of GCM-LGF.

All these experiments were performed using LGF purified from rats. We demonstrated that LGF purified from human subjects has the same chemical composition and equal biological activity [65]. Moreover, the use of LGF as a neural tissue regenerator is currently protected (patents: US 8,642,551 B2, 4 February 2014; CE, num. 09732019.6-1456, Ref. EP-883, April 2015; and US 14/140.014, 26 May 2015). Considering the possibility that LGF could be used as a therapeutic agent in PD, we have designed a strategy for LGF synthesis, and we aim to perform the corresponding preclinical assays before initiating human clinical studies. Our next objective is to find a company to collaborate with our group in these studies in order to synthesize LGF and to carry out the necessary preclinical and clinical studies to use LGF in human pathology.

## 5. Conclusions

Liver growth factor is an albumin–bilirubin complex that stimulates axonal growth in the striatum and protects DA neurons in the SN of 6-hydroxydopamine-lesioned rats. Our previous results suggested that these observed in vivo effects are mediated by microglia and/or astrocytes. In E14 rat mesencephalic glial cultures, LGF up-regulated MAPKs ERK1/2 and CREB phosphorylation in a small population of microglia. Moreover, LGF activated microglia and increased TNF-alpha levels in these glial cultures. The treatment of E14 midbrain neurons with glial-conditioned medium from LGF-treated glial cultures prevented the loss of DA neurons caused by 6-hydroxy-dopamine. This neuroprotective effect was not observed when GCM-LGF was applied in the presence of a blocking antibody of TNF-alpha activity. Altogether, our findings strongly suggest the involvement of microglia and TNF-alpha in the neuroprotective action of LGF on DA neurons observed in vitro.

## References

[1] Dauer W., Przedborski S. (2003). Parkinson’s disease: Mechanisms and models. Neuron.

[2] Savitt J.M., Dawson V.L., Dawson T.M. (2006). Diagnosis and treatment of Parkinson disease: Molecules to medicine. J. Clin. Investig..

[3] Chung Y.C., Ko H.W., Bok E., Park E.S., Huh S.H., Nam J.H., Jin B.K. (2010). The role of neuroinflammation on the pathogenesis of Parkinson’s disease. BMB Rep..

[4] Hald A., Van Beek J., Lotharius J. (2007). Inflammation in Parkinson’s disease: Causative or epiphenomenal?. Subcell. Biochem..

[5] Schulz J.B. (2005). Neuronal pathology in Parkinson’s disease. Cell Tissue Res..

[6] Pezzoli G., Zini M. (2010). Levodopa in Parkinson’s disease: From the past to the future. Expert Opin. Pharmacother..

[7] Fernandez-Espejo E. (2004). Pathogenesis of Parkinson’s disease: Prospects of neuroprotective and restorative therapies. Mol. Neurobiol..

[8] Yasuda T., Mochizuki H. (2010). Use of growth factors for the treatment of Parkinson’s disease. Expert Rev. Neurother..

[9] Ramaswamy S., Kordower J.H. (2009). Are growth factors the answer?. Parkinsonism Relat. Disord..

[10] Díaz-Gil J.J., Escartin P., Garcia-Canero R., Trilla C., Veloso J.J., Sanchez G., Moreno-Caparros A., Enrique de Salamanca C., Lozano R., Gavilanes J.G. (1986). Purification of a liver DNA-synthesis promoter from plasma of partially hepatectomized rats. Biochem. J..

[11] Díaz-Gil J.J., Gavilanes J.G., Sanchez G., Garcia-Canero R., Garcia-Segura J.M., Santamaria L., Trilla C., Escartin P. (1987). Identification of a liver growth factor as an albumin-bilirubin complex. Biochem. J..

[12] Díaz-Gil J.J., Sanchez G., Trilla C., Escartin P. (1988). Identification of biliprotein as a liver growth factor. Hepatology.

[13] Díaz-Gil J.J., Garcia-Monzon C., Rua C., Martin-Sanz P., Cereceda R.M., Miquilena-Colina M.E., Machin C., Fernandez-Martinez A., Garcia-Canero R. (2008). The anti-fibrotic effect of liver growth factor is associated with decreased intrahepatic levels of matrix metalloproteinases 2 and 9 and transforming growth factor beta 1 in bile duct-ligated rats. Histol. Histopathol..

[14] Díaz-Gil J.J., Munoz J., Albillos A., Rua C., Machin C., Garcia-Canero R., Cereceda R.M., Guijarro M.C., Trilla C., Escartin P. (1999). Improvement in liver fibrosis, functionality and hemodynamics in CCI4-cirrhotic rats after injection of the Liver Growth Factor. J. Hepatol..

[15] Gonzalo-Gobernado R., Calatrava-Ferreras L., Perucho J., Reimers D., Casarejos M.J., Herranz A.S., Jimenez-Escrig A., Diaz-Gil J.J., Bazan E. (2014). Liver growth factor as a tissue regenerating factor in neurodegenerative diseases. Recent Pat. CNS Drug Discov..

[16] Reimers D., Herranz A.S., Diaz-Gil J.J., Lobo M.V., Paino C.L., Alonso R., Asensio M.J., Gonzalo-Gobernado R., Bazan E. (2006). Intrastriatal infusion of liver growth factor stimulates dopamine terminal sprouting and partially restores motor function in 6-hydroxydopamine-lesioned rats. J. Histochem. Cytochem..

[17] Gonzalo-Gobernado R., Reimers D., Herranz A.S., Diaz-Gil J.J., Osuna C., Asensio M.J., Baena S., Rodriguez-Serrano M., Bazan E. (2009). Mobilization of neural stem cells and generation of new neurons in 6-OHDA-lesioned rats by intracerebroventricular infusion of liver growth factor. J. Histochem. Cytochem..

[18] Reimers D., Osuna C., Gonzalo-Gobernado R., Herranz A.S., Diaz-Gil J.J., Jimenez-Escrig A., Asensio M.J., Miranda C., Rodriguez-Serrano M., Bazan E. (2012). Liver growth factor promotes the survival of grafted neural stem cells in a rat model of Parkinson’s disease. Curr. Stem. Cell Res. Ther..

[19] Gonzalo-Gobernado R., Calatrava-Ferreras L., Reimers D., Herranz A.S., Rodriguez-Serrano M., Miranda C., Jimenez-Escrig A., Diaz-Gil J.J., Bazan E. (2013). Neuroprotective activity of peripherally administered liver growth factor in a rat model of Parkinson’s disease. PLoS ONE.

[20] F L.E., Tirolo C., Testa N., Caniglia S., Morale M.C., Marchetti B. (2010). Glia as a turning point in the therapeutic strategy of Parkinson’s disease. CNS Neurol. Disord. Drug Targets.

[21] Liberto C.M., Albrecht P.J., Herx L.M., Yong V.W., Levison S.W. (2004). Pro-regenerative properties of cytokine-activated astrocytes. J. Neurochem..

[22] McGeer P.L., McGeer E.G. (2008). Glial reactions in Parkinson’s disease. Mov. Disord..

[23] Teismann P., Schulz J.B. (2004). Cellular pathology of Parkinson’s disease: Astrocytes, microglia and inflammation. Cell Tissue Res..

[24] Batchelor P.E., Liberatore G.T., Wong J.Y., Porritt M.J., Frerichs F., Donnan G.A., Howells D.W. (1999). Activated macrophages and microglia induce dopaminergic sprouting in the injured striatum and express brain-derived neurotrophic factor and glial cell line-derived neurotrophic factor. J. Neurosci..

[25] Batchelor P.E., Porritt M.J., Martinello P., Parish C.L., Liberatore G.T., Donnan G.A., Howells D.W. (2002). Macrophages and Microglia Produce Local Trophic Gradients That Stimulate Axonal Sprouting Toward but Not beyond the Wound Edge. Mol. Cell Neurosci..

[26] Polazzi E., Contestabile A. (2002). Reciprocal interactions between microglia and neurons: From survival to neuropathology. Rev. Neurosci..

[27] Streit W.J. (2002). Microglia as neuroprotective, immunocompetent cells of the CNS. Glia.

[28] Suzuki T., Hide I., Ido K., Kohsaka S., Inoue K., Nakata Y. (2004). Production and release of neuroprotective tumor necrosis factor by P2X7 receptor-activated microglia. J. Neurosci..

[29] Remy S., Naveilhan P., Paille V., Brachet P., Neveu I. (2003). Lipopolysaccharide and TNFalpha regulate the expression of GDNF, neurturin and their receptors. Neuroreport.

[30] Schulte-Herbruggen O., Nassenstein C., Lommatzsch M., Quarcoo D., Renz H., Braun A. (2005). Tumor necrosis factor-alpha and interleukin-6 regulate secretion of brain-derived neurotrophic factor in human monocytes. J. Neuroimmunol..

[31] Cho S.G., Yi S.Y., Yoo Y.S. (2005). IFNgamma and TNFalpha synergistically induce neurite outgrowth on PC12 cells. Neurosci. Lett..

[32] Song J.H., Wang C.X., Song D.K., Wang P., Shuaib A., Hao C. (2005). Interferon gamma induces neurite outgrowth by up-regulation of p35 neuron-specific cyclin-dependent kinase 5 activator via activation of ERK1/2 pathway. J. Biol. Chem..

[33] Wong G., Goldshmit Y., Turnley A.M. (2004). Interferon-gamma but not TNF alpha promotes neuronal differentiation and neurite outgrowth of murine adult neural stem cells. Exp. Neurol..

[34] Fontaine V., Mohand-Said S., Hanoteau N., Fuchs C., Pfizenmaier K., Eisel U. (2002). Neurodegenerative and neuroprotective effects of tumor Necrosis factor (TNF) in retinal ischemia: Opposite roles of TNF receptor 1 and TNF receptor 2. J. Neurosci..

[35] Kuno R., Yoshida Y., Nitta A., Nabeshima T., Wang J., Sonobe Y., Kawanokuchi J., Takeuchi H., Mizuno T., Suzumura A. (2006). The role of TNF-alpha and its receptors in the production of NGF and GDNF by astrocytes. Brain Res..

[36] Díaz-Gil J.J., Majano P.L., Lopez-Cabrera M., Sanchez-Lopez V., Rua C., Machin C., Trilla C., Garcia-Canero R., Moreno-Otero R. (2003). The mitogenic activity of the liver growth factor is mediated by tumor necrosis factor alpha in rat liver. J. Hepatol..

[37] Singh J., Bowers L.D. (1986). Quantitative fractionation of serum bilirubin species by reversed-phase high-performance liquid chromatography. J. Chromatogr..

[38] Diaz Gil J.J., Rua C., Machin C., Cereceda R.M., Garcia-Canero R., de Foronda M., Perez de Diego J., Trilla C., Escartin P. (1994). Hepatic growth induced by injection of the liver growth factor into normal rats. Growth Regul..

[39] O’Malley E.K., Black I.B., Dreyfus C.F. (1991). Local support cells promote survival of substantia nigra dopaminergic neurons in culture. Exp. Neurol..

[40] Mena M.A., Pardo B., Paino C.L., De Yebenes J.G. (1993). Levodopa toxicity in foetal rat midbrain neurones in culture: Modulation by ascorbic acid. Neuroreport.

[41] Mena M.A., Casarejos M.J., Carazo A., Paino C.L., Garcia de Yebenes J. (1996). Glia conditioned medium protects fetal rat midbrain neurones in culture from L-DOPA toxicity. Neuroreport.

[42] Ungerstedt U. (1971). Stereotaxic mapping of the monoamine pathways in the rat brain. Acta Physiol. Scand. Suppl..

[43] Mena M.A., Casarejos M.J., Garcia de Yebenes J. (1999). The effect of glia-conditioned medium on dopamine neurons in culture. Modulation of apoptosis, tyrosine hydroxylase expression and 1-methyl-4-phenylpyridinium toxicity. J. Neural Transm. (Vienna).

[44] Rodriguez-Martin E., Canals S., Casarejos M.J., de Bernardo S., Handler A., Mena M.A. (2001). L-DOPA and glia-conditioned medium have additive effects on tyrosine hydroxylase expression in human catecholamine-rich neuroblastoma NB69 cells. J. Neurochem..

[45] Rodriguez-Martin E., Casarejos M.J., Bazan E., Canals S., Herranz A.S., Mena M.A. (2000). Nitric oxide induces differentiation in the NB69 human catecholamine-rich cell line. Neuropharmacology.

[46] Susin L. (2008). Capacidad antioxidante del Liver Growth factor: Estudios in Vitro e in Vivo en Ratas Espontáneamente Hipertensas. Ph.D. Thesis.

[47] Polazzi E., Altamira L.E., Eleuteri S., Barbaro R., Casadio C., Contestabile A., Monti B. (2009). Neuroprotection of microglial conditioned medium on 6-hydroxydopamine-induced neuronal death: Role of transforming growth factor beta-2. J. Neurochem..

[48] Ralay Ranaivo H., Wainwright M.S. (2010). Albumin activates astrocytes and microglia through mitogen-activated protein kinase pathways. Brain Res..

[49] Lee H.T., Chang Y.C., Tu Y.F., Huang C.C. (2010). CREB activation mediates VEGF-A’s protection of neurons and cerebral vascular endothelial cells. J. Neurochem..

[50] Mastroeni D., Grover A., Leonard B., Joyce J.N., Coleman P.D., Kozik B., Bellinger D.L., Rogers J. (2009). Microglial responses to dopamine in a cell culture model of Parkinson’s disease. Neurobiol. Aging.

[51] Saha R.N., Liu X., Pahan K. (2006). Up-regulation of BDNF in astrocytes by TNF-alpha: A case for the neuroprotective role of cytokine. J. Neuroimmune Pharmacol..

[52] Lai A.Y., Todd K.G. (2008). Differential regulation of trophic and proinflammatory microglial effectors is dependent on severity of neuronal injury. Glia.

[53] Liang J., Takeuchi H., Jin S., Noda M., Li H., Doi Y., Kawanokuchi J., Sonobe Y., Mizuno T., Suzumura A. (2010). Glutamate induces neurotrophic factor production from microglia via protein kinase C pathway. Brain Res..

[54] Shein N.A., Grigoriadis N., Horowitz M., Umschwief G., Alexandrovich A.G., Simeonidou C., Grigoriadis S., Touloumi O., Shohami E. (2008). Microglial involvement in neuroprotection following experimental traumatic brain injury in heat-acclimated mice. Brain Res..

[55] Kraft A.D., McPherson C.A., Harry G.J. (2009). Heterogeneity of microglia and TNF signaling as determinants for neuronal death or survival. Neurotoxicology.

[56] Taoufik E., Petit E., Divoux D., Tseveleki V., Mengozzi M., Roberts M.L., Valable S., Ghezzi P., Quackenbush J., Brines M. (2008). TNF receptor I sensitizes neurons to erythropoietin- and VEGF-mediated neuroprotection after ischemic and excitotoxic injury. Proc. Natl. Acad. Sci. USA.

[57] Schmitt K.R., Boato F., Diestel A., Hechler D., Kruglov A., Berger F., Hendrix S. (2010). Hypothermia-induced neurite outgrowth is mediated by tumor necrosis factor-alpha. Brain Pathol..

[58] Lian H., Zheng H. (2016). Signaling pathways regulating neuron-glia interaction and their implications in Alzheimer’s disease. J. Neurochem..

[59] Sagare A.P., Winkler E.A., Bell R.D., Deane R., Zlokovic B.V. (2011). From the liver to the blood-brain barrier: An interconnected system regulating brain amyloid-beta levels. J. Neurosci. Res..

[60] Thornalley P.J. (1998). Cell activation by glycated proteins. AGE receptors, receptor recognition factors and functional classification of AGEs. Cell Mol. Biol. (Noisy-le-grand).

[61] Park I.H., Yeon S.I., Youn J.H., Choi J.E., Sasaki N., Choi I.H., Shin J.S. (2004). Expression of a novel secreted splice variant of the receptor for advanced glycation end products (RAGE) in human brain astrocytes and peripheral blood mononuclear cells. Mol. Immunol..

[62] Wang Z., Li D.D., Liang Y.Y., Wang D.S., Cai N.S. (2002). Activation of astrocytes by advanced glycation end products: Cytokines induction and nitric oxide release. Acta Pharmacol. Sin..

[63] Martin H.L., Teismann P. (2009). Glutathione—A review on its role and significance in Parkinson’s disease. FASEB J..

[64] Sandhu J.K., Gardaneh M., Iwasiow R., Lanthier P., Gangaraju S., Ribecco-Lutkiewicz M., Tremblay R., Kiuchi K., Sikorska M. (2009). Astrocyte-secreted GDNF and glutathione antioxidant system protect neurons against 6OHDA cytotoxicity. Neurobiol. Dis..

[65] Diaz-Gil J.J., Gavilanes J.G., Garcia-Canero R., Garcia-Segura J.M., Santamaria L., Trilla C., Martin A.M., Guerra M.A., Garcia-Escandon F., Iniguez J.A. (1989). Liver growth factor purified from human plasma is an albumin-bilirubin complex. Mol. Biol. Med..

